# Supplementary material for: The treatment outcomes of epilepsy and its root causes in children attending at the University of Gondar teaching hospital: A retrospective cohort study, 2018
Source: PLoS One. 2020 Mar 12;15(3):e0230187. doi: 10.1371/journal.pone.0230187 (PMC7067446; doi:10.1371/journal.pone.0230187)
Supplement: Attachment — Submitted filename: Response to reviwers.docx [file pone.0230187.s002.docx]

**Point-by-point response** **to the comments**

Dear editor and reviewer, I want to use this opportunity to say thank you very much for your time to review our abstract. I found that your comments are very constructive and will add important values on our paper. I hope the quality of the manuscript will be increased after considering of the comments given by you. We tried to address all the comments given to us and included in the main manuscript document. The corrections made are given below as seen from the table.

Thank you again for your action!!

**Comments given by the reviewer and corrections taken**

**Reviewer 1(LOMPO Djingri Labodi)**

| **Section** | **Given comments** | **Response /reflection** |
| --- | --- | --- |
| Methodology | Operational definitions: Interest in adding a temporal criterion to evaluate the effectiveness of anti-epileptic treatment (epileptic seizure control), for example, at least 3 months after the start of treatment | Thank you for your clear comments.  Well controlled seizure: Maximum of one seizure episode in the last 3 months after the start of treatment.  Good control seizure: Maximum of three seizure episodes in the last 3 months after the start of treatment.  Poorly controlled seizure: Maximum of nine, and a minimum of four seizure episodes in the last three months after the start of treatment.  Uncontrolled seizure: Ten and above seizure episodes in the last three months after the start of treatment. |
| Results | Sociodemographic characteristics of patients • Table I: Well transcribing age groups: [5-10], [10-15], [15-18] | Thank you! We corrected the given comments in the clear and track changed manuscript. |
|  | • Table 2: review the multivariate logistic regression procedure to make the results more relevant: instead, refer to poor adherence and compare it with excellent and good adherence | We run logistic regression as per the given comments and narrated as below.  The likelihood of developing a successful treatment outcome in patients with excellent adherence (AOR=4. 51; 95%CI: 1.53, 13.42) was about 4.5 times higher than those with poor adherence (**table 3**). |
| Discussion | • Explain how poor compliance is a factor in therapeutic failure | The possible justification for this finding could be, when patients have a critical attention to the medication intake, they have the best treatment outcome of the epilepsy. However, if they fail to take their medication as per the physician prescribed (drug dose and frequency), they have the tendency of poor treatment outcome of epilepsy. |
|  | • There is also a need to explain how gender is a factor associated with the outcome of anti-epileptic treatment. | This could be explained by the fact that females give better due attention to every aspect of their life, and they might think everything critically and they might also afraid of the complications of missing the anti-epilepsy medications, which in fact might be associated with better success of treatment outcome of epilepsy. |

**Reviewer 2**

| **Section** | **Given comments** | **Response /reflection** |
| --- | --- | --- |
| All parts | 1.There are several grammatical errors which need to be addressed or use of an editing service. | We reviewed the manuscript with the help of reverso- grammerly software and the language expert. |
|  | 2. Some of the sentences are very long for example in the background section "In developing countries ......" | Epilepsy is the most common chronic neurological disorder which affects an estimated 10.5 million children worldwide, and 80% of them live in developing countries, often accompanied by physical and cognitive disability (1-3). Most children may have at least one epileptic seizure that is recommended for anti-epileptic drugs; which are prescribed according to episodes and severity of seizures (4). Studies are highly important that initiate to develop methods towards epilepsy treatment and to evaluate the effective strategies for the therapeutic purpose (5, 6). Various studies revealed that having different treatment protocols, inaccurate epilepsy diagnosis, inappropriate health care provider, lack of modern diagnostic technology, delay in seeking health care, and lack of knowledge were the identified factors of treatment outcome of epilepsy in developing countries (7, 8). Despite epilepsy is the crucial public health problem (9), there are scanty studies on the treatment outcomes of epilepsy in children in Ethiopia in general, and in the study area in particular. Therefore, this study was designed to determine the treatment outcomes of epilepsy and its predictors in children attending at the University of Gondar teaching hospital. |
|  | 3. In methods, this it is reported that this was a retrospective study, however details regarding this are missing, was it a chart review? in other instances it states it included study participants, I think clarity is needed here. | Yes! The study was a retrospective cohort study. And we went back for two years and we collected the data from the chart review and we got consent from the patients/ patient’s guardians. |
|  | 4. how was drug adherence determined? | The number of tablets the patient took, and the number of days the patient missed the anti-epileptic drugs during each of the follow up month was counted and put in percentage.  If the patient took more than 90% of his/her monthly medication, he/she had an excellent adherence; If the patient took more than 85% of his/her monthly medication, he/she had good adherence. Similarly, if he/she took less than 85% of his/her monthly medication, he/she had poor adherence. |
|  | 5. what do you mean by escalation phase of treatment? | Escalation phase is the phase at which the patient will take the lowest dose of the drug until the maximum recommended dose is reached or the maximum effect of the drug on the disease achieved.  Literally, it is the opposite of tapering; reducing the dose of the drug after the disease controlled. |
|  | 6. Why is the treatment response of children poor compared to adults? Is it due to adherence issues or type of medications being used? | The treatment response of children is poor compared to adults. This might be due to the children could forget the daily prescribed medication and may not forget to take. And also, mostly the child’s medication would be provided by the caregivers/ Guardians and as a result, it has the tendency to forget the daily medication. |
|  | 7. The authors do not report the root causes of treatment outcomes. | In this study, we reported ‘root causes of, treatment outcome’ as the factors associated with the treatment outcomes of anti-epilepsy medications. |

**Reviewer 3**

| **Section** | **Given comments** | **Response /reflection** |
| --- | --- | --- |
|  | The authors should rewrite the manuscript and use the appropriate grammar. | Thank you for your clear comments.  We reviewed the manuscript with the help of reverso- grammerly software and the language expert. |
|  | The outcome measure is not clearly stated. The "treatment outcomes" which is the purpose of this study does not come out of clearly and it is mixed up with the factors that affect the "treatment outcome" as defined by the authors | Thank you for your critical comments.  Treatment outcome: Patients who had a maximum of three seizure episodes in the 3 months after the start of anti-epileptic treatment, it was considered as having a **successful treatment outcome.** However, if the patient had four and above seizure episodes in the 3 months after the start of anti-epileptic treatment, he/she had **not successful treatment outcome.** |
