# Supplementary material for: The treatment outcomes of epilepsy and its root causes in children attending at the University of Gondar teaching hospital: A retrospective cohort study, 2018
Source: PLoS One. 2020 Mar 12;15(3):e0230187. doi: 10.1371/journal.pone.0230187 (PMC7067446; doi:10.1371/journal.pone.0230187)
Supplement: Attachment — Submitted filename: Response to Reviewers.docx [file pone.0230187.s003.docx]

Response to Reviewers

Dear editors

Great greetings! First, I would like to say thank you for your contribution for the improvement of this research paper due to the previous revision. As we see from the email of editorial office we see many things which are improved and some of the comments need our actions to do. Here we attached this activity performed.

Sincerely

Agumas Fentahun

Comments needs action

| S.no | Comment | Response | Remark |
| --- | --- | --- | --- |
| 1. | It will be necessary to remove "the educational status of caregiver | We remove it |  |
| 2. | This is obviously an improvement on the earlier manuscript. But the author/s makes a lot a grammar error that makes the flow of the information being presented inappropriate at times and difficult to read. I recommend the use of the English editors to help the author’s message come out clearly | We used gramerly applications, in addition, we invited for English language editors and rewrite it. |  |
|  |  |  |  |
